# Comparative Analysis of *Xenorhabdus koppenhoeferi* Gene Expression during Symbiotic Persistence in the Host Nematode

**DOI:** 10.1371/journal.pone.0145739

**Published:** 2016-01-08

**Authors:** Ruisheng An, Parwinder S. Grewal

**Affiliations:** Department of Entomology and Plant Pathology, University of Tennessee, 2505 E. J. Chapman Drive, Knoxville, TN, 37996, United States of America; James Hutton Institute, UNITED KINGDOM

## Abstract

Species of *Xenorhabdus* and *Photorhabdus* bacteria form mutualistic associations with *Steinernema* and *Heterorhabditis* nematodes, respectively and serve as model systems for studying microbe-animal symbioses. Here, we profiled gene expression of *Xenorhabdus koppenhoeferi* during their symbiotic persistence in the newly formed infective juveniles of the host nematode *Steinernema scarabaei* through the selective capture of transcribed sequences (SCOTS). The obtained gene expression profile was then compared with other nematode-bacteria partnerships represented by *Steinernema carpocapsae—Xenorhabdus nematophila* and *Heterorhabditis bacteriophora—Photorhabdus temperata*. A total of 29 distinct genes were identified to be up-regulated and 53 were down-regulated in *X*. *koppenhoeferi* while in *S*. *scarabaei* infective juveniles. Of the identified genes, 8 of the up-regulated and 14 of the down-regulated genes were similarly expressed in *X*. *nematophila* during persistence in its host nematode *S*. *carpocapsae*. However, only one from each of these up- and down-regulated genes was common to the mutualistic partnership between the bacterium *P*. *temperata* and the nematode *H*. *bacteriophora*. Interactive network analysis of the shared genes between *X*. *koppenhoeferi* and *X*. *nematophila* demonstrated that the up-regulated genes were mainly involved in bacterial survival and the down-regulated genes were more related to bacterial virulence and active growth. Disruption of two selected genes *pta* (coding phosphotransacetylase) and *acnB* (coding aconitate hydratase) in *X*. *nematophila* with shared expression signature with *X*. *koppenhoeferi* confirmed that these genes are important for bacterial persistence in the nematode host. The results of our comparative analyses show that the two *Xenorhabdus* species share a little more than a quarter of the transcriptional mechanisms during persistence in their nematode hosts but these features are quite different from those used by *P*. *temperata* bacteria in their nematode host *H*. *bacteriophora*.

## Introduction

Mutualistic associations between entomopathogenic nematodes *Steinernema* and *Heterorhabditis* spp. and their symbiotic bacteria *Xenorhabdus* and *Photorhabdus* spp. represent one of the best-developed systems for the study of animal-microbe symbiosis [1–3]. *Photorhabdus* cells colonize the proximal region of the gut of *Heterorhabditis* infective juvenile [4], but colonization of *Xenorhabdus* bacteria in *Steinernema* infective juveniles is restricted to a specialized intestinal receptacle [5–7]. Although *Heterorhabditis* and *Steinernema* belong to phylogenetically distant nematode clades, their symbiotic bacteria *Xenorhabdus* and *Photorhabdus* are more closely related to each other [8] and share very similar life histories [9]. The bacteria promote their own transmission among insects by using the nematode infective juvenile as a vector whereas the nematode uses the bacteria as food source [10–12]. When the infective juveniles invade a susceptible insect host, they migrate into the hemolymph to release the symbiotic bacteria. The bacteria appear to be defecated from the anal opening in the case of *Steinernema* [13] and regurgitated from the mouth of *Heterorhabditis* infective juvenile [14]. The bacteria multiply in the hemolymph and produce a plethora of biomolecules [15,16], killing the insect and converting the cadaver into a food source suitable for nematode growth and reproduction. After depleting the insect cadaver the bacteria recolonize the developing infective juveniles that exit the depleted cadaver in search of a new host, thus ensuring their own transmission to a new insect host using the nematode as a vector [1].

Previous investigation of *Photorhabdus luminescens* suggests that the mutualism is initiated when the bacteria express maternal adhesion fimbriae and adhere to the nematode intestine [17]. The bacteria are maternally transmitted to infective juveniles developing inside the mother’s body through a series of steps involving invasion and intracellular growth [18]. In this process, *Photorhabdus* bacteria switch from pathogenic variants to small-cell variants during initiation of the mutualism and then switch back to pathogenic form in the infective juvenile intestine to arm the nematodes for the next infection cycle [18]. In the *Steinernema*—*Xenorhabdus* symbiosis, it appears that a few bacterial cells initially colonize and remain in the nematode pharyngeal-intestinal valve before finally occupying and filling the vesicle according to a study of *X*. *nematohila* [19]. Nil factors A, B and C encoded on a symbiosis region specific to *X*. *nematophila* are independently necessary for this initiation process [19,20]. This symbiosis region seems also sufficient to confer entry into the nematode vesicle on otherwise non-colonizing *Xenorhabdus* species, albeit with lower levels of colonization than *X*. *nematophila* [19,21].

Mutualistic association protects the bacteria from the external environment while the host infective juveniles endure in soil without feeding for several months in search of a suitable insect host [22]. Fitness costs may be incurred by the nematode due to the provision of nutrition to the bacteria considering that axenic infective juveniles survive longer than colonized ones [23] and that bacterial mutants defective in the synthesis of some essential amino acids (e.g. histidine and serine) exhibited normal colonization efficiency in the infective juveniles [24,25]. Nevertheless, the stored energy reserves of infective juveniles are limited [22], and the number of bacterial cells in the nematode decreases progressively over time [25–27]. Therefore, both players need to evolve some kind of strategies to reduce the nutritional dependence of the bacteria on the nematode for better survival of the partnership before a suitable insect host is found. Recent evidence shows that carrying an intermediate number of bacterial cells maximizes their survivorship in the infective juveniles although carrying more provides higher parasitic success for the nematode [28]. It is further believed that such evolutionary trade-offs could be governed by molecular underpinning [28]. In fact, we previously discovered that *Photorhabdus temperata* cells slow down their growth and metabolism during persistence in the nematode partner *H*. *bacteriophora* compared to that in culture based on the changes in the bacterial transcriptional profile [27]. The term "persistence" used here refers to a survival strategy or phenotype wherein at least a small population of the symbiotic bacteria is maintained in the enduring nematode infective juvenile [29].

Comparative analysis displays genomic convergence in *Xenorhabdus* species and divergence between *Xenorhabdus* and *Photorhabdus* [30]. The molecular and genetic divergence is underscored by the differences in sites of bacterial transmission and colonization in the infective juvenile nematodes between the *Photorhabdus-Heterorhabditis* and *Xenorhabdus-Steinernema* systems [30]. So far, no overlap in genetic determinants essential for colonization of their nematode hosts has been observed between the two bacterial genera [30]. Therefore, we hypothesized that *Xenorhabdus* and *Photorhabdus* have independently evolved mutualistic associations with nematodes and they use different molecular mechanisms to persist in their respective nematode hosts. To test this hypothesis we first assessed gene expression of *Xenorhabdus koppenhoeferi* in *Steinernema scarabaei* infective juveniles and then compared it to other bacterial transcriptional profiles in the more widely studied mutualistic relationships of *X*. *nematophila*—*S*. *carpocapsae* and *P*. *temperata*—*H*. *bacteriophora*. Data for this comparison were obtained for *X*. *nematophila* also by SCOTS procedure in this study and for *P*. *temperata* from our previous study [27]. This led to the identification of gene expression signatures shared by these bacteria during persistence in their nematode hosts. Through mutational analyses, we further characterized the role of the shared gene-expression signature in the persistence of *Xenorhabdus* in the enduring *Steinernema* infective juveniles.

## Materials and Methods

### Nematodes and bacteria

The nematode *Steinernema scarabaei* strain AMK001 was originally obtained from Dr. Albrecht M. Koppenhöfer (Rutgers University, New Brunswick, New Jersey) and *Steinernema carpocapsae* strain All was obtained from Biosys (Palo Alto, California). The nematodes were cryopreserved in liquid nitrogen as described previously [31,32]. For the experiments in this study, the nematodes were cultured at 25°C by infecting the last instars of the Japanese beetle *Popillia japonica* (in case of *S*. *scarabaei*) or last instars of the wax worm *Galleria mellonella* (in case of *S*. *carpocapsae*). The bacteria *X*. *koppenhoeferi* (the symbiont of *S*. *scarabaei*) and *X*. *nematophila* (the symbiont of *S*. *carpocapsae*) were isolated from their host nematodes following the protocol described by Akhurst [33]. As *X*. *koppenhoeferi* could not be grown on the regular media like LB and Nutrient, the bacteria were routinely cultured in Brain Heart Infusion (BHI) medium at 25°C unless otherwise mentioned. To support *S*. *carpocapsae* growth, the bacteria *X*. *nematophila* were also grown on lipid agar (0.8% nutrient broth, 0.5% yeast extract, 0.2% MgCl_2_, 0.7% corn syrup, 0.4% corn oil, and 1.5% Bacto Agar) and incubated for 24 h before addition of nematodes [34]. M9 minimal medium [35] [12.8 g/L Na_2_HPO_4_∙7H_2_O, 3.0 g/L KH_2_PO_4_, 0.5 g/L NaCl, 1.0 g/L NH_4_Cl, 2.6 g/L (NH_4_)_2_SO_4_, 1.0 mM MgSO_4_, 100 μM CaCl_2_, 3 nM (NH_4_)_6_Mo_7_O_24_∙4H_2_O, 0.4 mM H_3_BO_3_, 30 nM CoCl_2_∙6H_2_O, 10 nM CuSO_4_∙5H_2_O, 80 nM MnCl_2_∙4H_2_O, 10 nM ZnSO_4_∙7H_2_O and 5 nM FeSO_4_∙7H_2_O] was used for phenotypic characterization of the bacterial mutants. Glucose (25 mM) or acetate (25 mM) was used as carbon source for the minimal medium with an attempt to understand the relevance and importance of certain genes identified in this study to the bacterial central metabolism. For anaerobic growth, the media were additionally supplemented with 50 mM fumarate as an electron acceptor, boiled to drive off oxygen, covered with sterilized mineral oil, and cooled down 30 min before inoculation of the bacterial cells [36]. All the bacterial cultures and growth were maintained in dark throughout the study.

### Selective capture and enrichment of differentially transcribed sequences

Gene expression of *X*. *koppenhoeferi* in the infective juvenile of *S*. *scarabaei* was profiled through selective capture of transcribed sequences (SCOTS) procedure as described in our previous study [27]. In short, total RNA was extracted using TRIzol reagent (Invitrogen) from over-night cultured *X*. *koppenhoeferi* cells grown in BHI broth at 25°C and from surface-sterilized freshly produced *S*. *scarabaei* infective juveniles that were collected from the nematode-infected *P*. *japonica* cadavers using White traps [37] held at 25°C for 15–16 days. Since the White traps were observed every other day, the infective juveniles were collected between 3 and 5 days of the initiation of emergence. The total RNAs were first converted to single-stranded cDNAs by random priming with 5'-end tagged primers ST0N (for RNA samples from over-night cultured cells) and ST18N (for RNA samples from infective juveniles) (See S1 Table for the primer sequences) and then made double-stranded with Klenow fragment (NEB, Beverly, MA) as previously described [38]. After amplification with the respective tag sequences ST0 and ST18, the cDNAs were normalized by hybridization to biotin-labeled *X*. *koppenhoeferi* genomic DNA that had been blocked beforehand with an amplified bacterial rRNA operon. Bacterial cDNAs that hybridized to biotin-labeled genomic DNA were retained by binding hybrids to streptavidin-coated magnetic beads (Dynal, Bethlehem, PA) according to the manufacturer's instructions. This normalization step resulted in bacterial cDNAs (representing the bacterial mRNA transcripts) being exclusively captured from the cDNA mixture and more details about this normalization step had been described in previous reports [27,39–41]. Three independent cDNA samples from the over-night cultured cells or infective juveniles were normalized separately, pooled and considered as bacterial *in vitro* or *in vivo* cDNA libraries. Purification of the bacterial cDNAs apart from the nematode cDNAs after each cycle of normalization was monitored through PCR detection of the eukaryotic 18S rRNA gene with primers 18SF and 18SR [27]. The bacterial genes preferentially up-regulated in the infective juvenile relative to the *in vitro* culture were enriched by subtractive hybridization of the normalized *in vivo* cDNAs to the biotin-labeled bacterial genomic DNA that had been pre-hybridized with the rRNA operon and the normalized *in vitro* cDNAs. Vice versa, bacterial genes specifically down-regulated in the infective juvenile were enriched by subtractive hybridization of the normalized *in vitro* cDNAs to the biotin-labeled bacterial genomic DNA that had been pre-hybridized with the rRNA operon and the normalized *in vivo* cDNAs. The prokaryotic housekeeping gene gyrase A (*gyrA*) was used as a control to ensure that only differentially expressed genes were captured after enrichment. This was done through PCR detection of the *gyrA* genes with primers gyrAF and gyrAR after each cycle of enrichment [27]. The enriched bacterial cDNAs were cloned into an original TA cloning vector to construct libraries representing bacterial genes up- or down-regulated in the infective juvenile relative to the *in vitro* culture.

### Analysis of bacterial genes differentially expressed in the nematode host

A total of 360 clones were randomly selected from the constructed libraries for Southern dot-blot screening which has been used as a confirmatory test in most SCOTS-based research [39,42]. Individual clones from the enriched libraries were PCR amplified by M13 primers and hybridized with the probes made from the normalized cDNAs using PCR DIG Probe Synthesis kit (Roche). Successful hybridization was detected using anti-digoxigenin-HRP conjugate and Amersham ECL Plus western blotting detection reagents (GE Healthcare Bio-Sciences Corp). Individual clones that hybridized to either the probe made from the normalized *in vivo* cDNAs or the probe made from the normalized *in vitro* cDNAs were sequenced. The resulting sequences were analyzed by BLAST algorithms in GenBank. Average number of clones for each identical gene was expected to occur at least twice in the sequenced samples. Rarefaction analysis was used to estimate coverage of the enriched cDNA libraries for the identified genes as described previously [27,43]. In case of low coverage, more clones would be selected from the enriched libraries for Southern dot-blot screening and sequencing analysis. Functions of the identified genes were predicted and assigned using BioCyc databases [44]. The gene expression profile generated by the SCOTS approach was validated through Quantitative real-time PCR (qPCR) on the representative genes which were randomly selected but based on the success of primer design from the identified sequences. Relative fold change of gene expression was calculated from ΔΔCt using 16s rRNA gene as an internal control as previously described [45].

### Screening of starvation response genes

Since food may be limited for the bacteria during persistence in the non-feeding nematode infective juvenile, we distinguished bacterial genes which were differentially regulated due to general starvation. The above identified bacterial transcripts were screened with a stationary-phase cDNA library by Southern dot-blot hybridization. In brief, anaerobic and starvation conditions that may exist within the infective juvenile were simulated by culturing the bacteria under static growth conditions for 30 days at 25°C in sealed test tubes to reach a nutrient-depleted stationary phase [27,46]. Subsequently, total RNA was isolated and stationary-phase cDNA library was constructed with primers SCOT110N and SCOT110 following the SCOTS procedure. The DIG-labeled probe was prepared from the normalized stationary-phase cDNA library and used to screen the above identified bacterial transcripts by Southern dot-blot hybridization as described above.

### Cross-species comparative hybridization

We previously profiled gene expression of the bacteria *P*. *temperata* in the nematode host *H*. *bacteriophora* [27]. With the data obtained from *X*. *koppenhoeferi* in this study, we further compared gene expression between these two bacteria. Cross-species comparative hybridization as described by An et al. [16] was applied to determine how the gene expression was shared between *P*. *temperata* and *X*. *koppenhoeferi* or specific to either species. We first assessed genomic presence of the differentially expressed transcripts in the two bacteria. Transcripts identified from one bacterium were individually screened by hybridization to the sonicated biotin-labeled genomic DNA of the other, and the genomic presence was detected by a positive PCR amplification using the tag sequence as described above. The transcripts with sequences present in both bacterial genomes were singled out to evaluate the exclusivity of gene expression to *X*. *koppenhoeferi* or *P*. *temperata*. The selected transcripts differentially expressed in one bacterium were individually screened by hybridization to the sonicated biotin-labeled genomic DNA of the other that had been pre-hybridized with the respective rRNA operon and normalized *in vitro* cDNAs. A shared gene expression signature was indicated by a positive PCR amplification of the hybrids using the tag sequence.

### Shared gene expression signature

In addition, we were interested in the gene expression signature shared by different *Xenorhabdus* species during persistence in their respective nematode hosts. We first examined whether the identified *X*. *koppenhoeferi* genes were present in other *Xenorhabdus* and *Photorhabdus* genomes. Each identified *X*. *koppenhoeferi* gene sequence was analyzed by a blastn search against currently available genomes of *Xenorhabdus nematophila*, *Xenorhabdus bovienii*, *Photorhabdus luminescens*, and *Photorhabdus asymbiotica*. The discontiguous megablast for more dissimilar sequences was used in the blastn search with default general parameters of expect threshold (10), match/mismatch scores (2,-3), and gap costs (existence: 5; Extension: 2). Gene sequences present in the *X*. *nematophila* genome were selected for screening to detect expression signatures shared with *X*. *koppenhoeferi* during persistence in the host nematode. Following the selective capture and enrichment of differentially transcribed sequences procedure described above *X*. *nematophila in vivo* and *in vitro* cDNA libraries representing up- and down-regulated genes during persistence in the *S*. *carpocapsae* infective juvenile were prepared. Clones of the selected genes based on the genomic presence were individually screened by dot-blot hybridization to digoxigenin-labeled probes made from the *X*. *nematophila* cDNA libraries. The probes made from the *in vivo* and *in vitro* cDNA libraries were used to screen the selected genes up- and down-regulated in *X*. *koppenhoeferi*, respectively. The hybridization between the probes and the selected genes was performed at 65°C for 24 h. The positive hybridization indicating a shared gene expression signature between the two bacterial species was detected by anti-digoxigenin-HRP conjugate and Amersham ECL Plus western blotting reagents.

### Network analysis of the genes with shared expression signature

Functional annotation networks were produced using Pathway Studio software (Ariadne Genomics Inc., Rockville, MD) to predict the biological processes involved with the shared gene expression signature. This software uses MedScan, a literature mining program based on Natural Language Processing Technology to extract connectivity or relationships (binding, regulation, transportation or modification, etc.) among genes and delineate functionally-related networks from the published literature. By default, each relationship between the genes in the network is supported by at least one reference, and the created network can demonstrate various cellular processes and pathways in which the gene products are involved [47]. Here, the network was generated initially using the genes with shared expression signature, and then expanded with other relevant genes that are connected to the initial genes.

### Construction of deletion mutants

The insertion-deletion mutants of two selected genes (*pta* and *acnB*) with a shared expression signature were generated in *X*. *nematophila* to investigate their relevance to the symbiotic association of *Xenorhabdus* bacteria with *Steinernema* nematodes. The mutants were constructed in *X*. *nematophila* rather than in *X*. *koppenhoeferi* for two reasons. First, according to a previous report [48] and our preliminary tests, mass production of *S*. *scarabaei* is inconsistent on its symbiotic bacteria *X*. *koppenhoeferi* and in insects (the tested insects included European chafer *Rhizotrogus majalis*, Japanese beetle *P*. *japonica*, Masked chafer *Cyclocephala borealis*, and wax moth *G*. *mellonella*). Thus, it was deemed to be unreliable to use *X*. *koppenhoeferi—S*. *scarabaei* partnership for *in vitro* studies. Second, the nematode *S*. *carpocapsae* can be easily cultured *in vitro* with *X*. *nematophila* cells and gene knockouts and subsequent *in vitro* symbiosis assays had been successfully performed in this species [49]. To make the insertion-deletion mutation, a Cm^R^-cassette obtained from a template plasmid pAKCYC184 (BioLabs) was inserted through fusion PCR into the target gene of *X*. *nematophila* [18]. The used primers are listed in S1 Table in which P1 and P2 were for amplification of the upstream fragment of the target gene, P5 and P6 were for amplification of downstream fragment of the target gene, and P3 and P4 were used to amplify the insertion Cm^R^-cassette. The fused product was introduced into electrocompetent *X*. *nematophila* cells that had been engineered with a Red helper plasmid pKD119 (Yale Genetic Stock Center) to increase homologous recombination efficiency [50]. The electrocompetent *Xenorhabdus* cells were prepared freshly from fast growing culture as described earlier [51,52]. Briefly, 100 ml LB medium supplemented with 0.1% L-arabinose was inoculated with 100 μl over-night cultured *X*. *nematophila* cells and incubated at 25°C with shaking to early exponential phase (OD600 = 0.4). Bacterial growth was quickly stopped by chilling the culture flask in ice for 1 h. Bacterial cells were collected by centrifugation at 4,000 rpm for 5 min at 4°C. The pellets were re-suspended and sequentially washed three times at low temperature in freshly prepared ice-cold SH buffer (5% sucrose and 10mM HEPES in MilliQ water). The pellets were finally re-suspended in 500 μl SH buffer and stored on ice. For electroporation, the spliced DNA product as prepared above was mixed with 50 μl of freshly prepared ice-cold electrocompetent cells in a 0.2 cm cuvette (Bio-Rad) and pulsed at 2.5 kV with a MicroPulser (Bio-Rad). The desired recombinant was selected on LB antibiotic agar plate with chloramphenicol (34 μg/ml) and confirmed with PCR amplification.

### Complementation of the created mutants

To complement the gene mutation, the full length of the target gene containing its predicted native promoters was amplified with the primers listed in S1 Table (pta-FFL and pta-RFL for *pta* gene; acnB-FFL and acnB-RFL for *acnB* gene). The amplified products were digested with BamHI and XbaI restriction enzymes, followed by ligation into a mobile plasmid pJB861 (NBRP). To avoid the frequency of pulsed electrical stimulations which may cause potential damage to the created mutants, the pJB861 plasmid vectoring the target gene was chemically transformed into *E*. *coli* S-17.1 and conjugated into the desired *X*. *nematophila* mutant according to the method described previously [52]. The conjugant was selected on the antibiotic LB agar plate with kanamycin (50 mM), chloramphenicol (34 μg/ml) and 3-mythylbenzoic acid (2 mM), and confirmed with catalase reaction and PCR amplification.

### *In vitro* phenotypic characterization of the mutants

Cultural properties of the mutants were observed for possible phenotypic defects. The examined phenotypic traits included biofilm formation, motility, cell growth, survival and viability as these properties have been suggested to be important to the bacteria during persistence with their nematode partner [27,36]. Biofilm formation was assayed by the ability of the bacterial cells adhering to the wells of the polystyrene micro-plates as described in our previous study [36]. The bacterial motility was examined by cell diffusion in semi-solid motility test medium (per 1 L distilled water: 18.5 g BHI, 3 g agar, and 0.04 g 2, 3, 5-triphenyltetrazolium chloride). The bacterial growth was measured by optical density of the cell suspension at 600 nm (OD 600). The bacterial viability was evaluated by plating the serial dilution of cells on BHI agar, counting the colonies after 24-h incubation at 25°C, and then calculating the colony-forming units (CFU). All assays were performed in triplicate using three independent cultures of the test bacteria for a minimum of two times. The data were subjected to analysis of variance and significant differences were determined by multiple *t* tests at p < 0.05 with comparison to the wild-type strain.

### Nematode colonization assays

Effects of mutations on symbiotic interactions between *X*. *nematophila* and *S*. *carpocapsae* were assessed through the ability of the bacteria in colonizing the infective juveniles. Each *X*. *nematophila* strain was grown on lipid agar plate at 25°C for 24 h [34]. In the meantime axenic *S*. *carpocapsae* eggs were isolated from adult female nematodes according to a previously described procedure [53]. Approximately 100 axenic nematode eggs were propagated on each bacterial plate and incubated at 25°C. Nematode development was monitored daily until the production of the infective juveniles. The number of bacterial cells in the infective juveniles was determined following a previously described grinding procedure with some modifications [54]. Briefly, about 1000 infective juveniles sterilized with 0.1% merthiolate solution were crushed using an autoclaved micro pestle. The serial dilutions were spread on BHI agar, and bacterial colonies were counted following incubation at 25°C for 3 days. The assays were performed in triplicate and the data were subjected to analysis of variance with significant difference tests at p = 0.05.

## Results

### *X*. *koppenhoeferi* genes differentially regulated in the nematode host

To identify *X*. *koppenhoeferi* genes significantly up- or down-regulated in the nematode's intestinal vesicle, the bacteria-nematode cDNA mixture was normalized by the SCOTS approach resulting in separation of bacterial mRNA transcripts from the ribosomal and nematode transcripts. Detection of the eukaryotic 18S rRNA gene in the *in vivo* cDNA population before but not after normalization suggested that bacterial cDNAs were successfully purified from the nematode cDNAs. Detection of the prokaryotic *gyrA* gene in all other cDNA populations except the enriched cDNAs indicated that only differentially expressed transcripts were isolated after enrichment. Among the 360 randomly screened clones, 150 out of 192 from the up-regulated library and 147 out of 168 from the down-regulated library produced strong hybridization signals in Southern dot blot analysis. The clones with strong signals were considered to be reliable transcripts specifically regulated in *X*. *koppenhoeferi* during persistence in *S*. *scarabaei* infective juveniles. Sequencing of the clones with strong signals identified 82 distinct transcripts including 29 up-regulated and 53 down-regulated genes (Table 1). These gene sequences were deposited into the GenBank/NCBI with the accession numbers of JZ717596-JZ717677. During sequencing, each up- or down-regulated gene was identified at least twice from the clones. According to the rarefaction curves (S1 Fig), saturation was achieved for both the enriched *in vivo* and *in vitro* cDNA libraries. This indicated that the randomly selected clones represented the enriched cDNA libraries and most of the genes in the constructed libraries had been identified. Screening with the normalized cDNA library prepared from the bacteria grown *in vitro* under stationary-phase starvation conditions suggested that almost half of the identified genes (11 up- and 26 down-regulated) were differentially regulated due to general starvation and thus the rest of the genes were regulated specifically in response to the vesicle environment in the infective juvenile (Table 1). The patterns of gene expression determined by SCOTS were further validated by the qPCR analysis which showed consistent results on the 14 selected representative genes in *X*. *koppenhoeferi* (S2 Fig).

**Table 1:** Differentially expressed *Xenorhabdus koppenhoeferi* genes in *Steinernema scarabaei* infective juveniles.

Class	Gene -Possible function	Id/Sim%	Span	E-value
**Cell surface**	***pilT* (-)** -Pilus retraction ATPase PilT (*Serratia proteamaculans*, Spro_4029; *P*. *luminescens*, plu1181)	73/87	172	2e-121
	***rhs* (-)** -Extracellular solute-binding protein (*Photorhabdus luminescens*, plu2212)	69/80	142	6e-14
**Regulation**	***flhC* (+)** -Flagellar transcriptional activator (*Xenorhabdus nematophila*, XNC1_1626)	97/110	110	1e-33
	***hpcR* (+)** -Homoprotocatechuate degradation operon regulator (*X*. *nematophila*, XNC1_0824)	208/233	233	1e-83
	***iscR* (+)** -Transcriptional regulator (*X*. *nematophila*, XNC1_3297)	148/166	166	3e-57
	***lysR* (+)** -Transcriptional regulator/Translation inhibitor protein (*Erwinia carotovora*, ECA3134)	78/89	171	4e-23
	***lepA*** **(-)** -GTP-binding protein LepA (*P*. *luminescens*, plu3342)	86/92	255	1e-36
	***asmA* (-)** -Unlisted suppressor of ompF assembly mutants (*X*. *nematophila*, XNC1_1500)	145/174	216	3e-45
	***tldD*** **(-)** -Suppressor of inhibitory activity of the carbon storage regulator (*P*. *luminescens*, plu4064)	85/92	276	3e-19
	***tyrR* (-)** -Transcriptional regulator (*P*. *luminescens*, plu2580)	89/93	144	1e-20
**Stress response**	***cutC* (-)** -Copper homeostasis protein (*P*. *luminescens*, plu2101)	64/85	143	1e-09
	***grsD*** **(+)** -Linear gramicidin synthetase subunit D (*Pseudomonas aeruginosa*, PSPA7_2858)	57/74	166	2e-10
	***ppiD*** **(-)** -Peptidyl-prolyl cis-trans isomerase D (PPIase D) (*P*. *luminescens*, plu3865)	62/84	98	1e-04
**Nucleic acid modification**	***deaD*** **(+)** -Putative DEAD box family DNA helicase (*X*. *nematophila*, XNC1_2181)	237/268	268	1e-94
	***XK_scots01* (+)** -Putative helicase (*Vibrio parahaemolyticus*, VP1072)	67/79	171	7e-14
	***XK_scots02* (+)** -Putative integrase/recombinase (*X*. *nematophila*, XNC1_2234)	116/140	140	4e-35
	***XK_scots03*** **(+)** -Transposase (*X*. *nematophila*, XNC1_3131)	127/146	148	8e-45
	***XK_scots04*** **(+)** -Insersion sequence (*X*. *nematophila*, XNC1_1660)	105/125	209	3e-32
	***XK_scots05* (+)** -Putative resolvase (*Agrobacterium* sp, AF335479)	85/92	177	2e-21
	***XK_scots06*** **(+)** -IS2000-family transposase (*X*. *nematophila*, XNC1_0251)	177/182	182	3e-83
	***XK_scots07*** **(+)** -Transposase (*X*. *nematophila*, XNC1_0557)	221/223	224	9e-110
	***XK_scots08*** **(+)** -Putative transposase (*X*. *nematophila*, XNC1_2420)	273/282	283	8e-131
	***XK_scots09*** **(+)** -IS element transposase (*X*. *nematophila*, XNC1_2014)	177/188	187	3e-77
	***heaP*** **(-)** -ATP-dependent helicase (*P*. *luminescens*, plu0615)	90/95	140	4e-20
	***intB* (-)** -Integrase (*X*. *nematophila*, XNC1_3443)	116/125	166	6e-47
	***rep* (-)** -ATP-dependent DNA helicase (*Yersinia pseudotuberculosis*, YPTS_0175; *P*. *luminescens*, plu4667)	84/90	166	1e-21
	***ruvA*** **(-)** -Holliday junction DNA helicase motor protein (*P*. *luminescens*, plu2111)	90/97	174	1e-24
	***xni*** **(-)** -5'-3' exodeoxyribonuclease IX (*P*. *luminescens*, plu0660)	81/90	140	8e-17
**Transport**	***kefC* (+)** -Potassium efflux system protein (*X*. *nematophila*, XNC1_0944)	110/125	132	8e-39
	***ompX* (+)** -Outer membrane autotransporter barrel domain (*S*. *proteamaculans*, Spro_4737)	60/78	219	4e-17
	***yjgQ* (+)** -Predicted permeases (*P*. *luminescens*, plu4479)	61/80	177	2e-15
	***cyoD*** **(-)** -Cytochrome D ubiquinol oxidase (*P*. *luminescens*, plu3878)	68/79	180	1e-17
	***flhA* (-)** -Flagellar export apparatus (*P*. *luminescens*, plu1896)	82/93	144	7e-07
	***XK_scots10* (-)**–Putative haemolysin co-regulated protein (*X*. *nematophila*, XNC1_3435)	130/154	211	2e-41
	***nqrC*** **(-)** -Na(+)-translocating NADH:quinone reductase, chain C (*P*. *luminescens*, plu1197)	79/89	188	5–32
	***nqrF*** **(-)** -Na (+)-translocating NADH:ubiquinone oxireductase subunit F (*P*. *luminescens*, plu1201)	98/100	162	6e-28
	***potD* (-)** -Spermidine/putrescine ABC transporter (*Enterobacter sakazakii*, ESA_02223)	75/88	133	4e-17
	***secD*** **(-)** -Protein-export membrane protein (*P*. *luminescens*, plu3902)	88/92	200	1e-26
**Metabolism**	***aceE*** **(+)** -Pyruvate dehydrogenase E1 component (*X*. *nematophila*, XNC1_1080)	142/157	157	3e-56
	***acnB* (+)** -Aconitate hydratase (*X*. *nematophila*, XNC1_1090)	212/245	245	2e-79
	***pta*** **(+)** -Phosphotransacetylase (*X*. *nematophila*, XNC1_2801)	175/199	199	5e-67
	***xpsD* (+)** -Peptide synthetase (*Xenorhabdus bovienii*, XBJ1_2152)	209/297	347	2e-33
	***accC*** **(-)** -Acetyl-CoA carboxylase, biotin carboxylase (*Haemophilus somnus*, HSM_1606; *P*. *luminescens*, plu4075)	84/93	193	9e-31
	***atoA* (-)** -Acetate/3-ketoacid CoA transferase; coenzyme A transferase (*Shewanella sediminis*, Ssed_3536)	44/64	176	6e-07
	***coaA*** **(-)** -Pantothenate kinase (*P*. *luminescens*, plu4731)	79/88	204	1e-25
	***dapF* (-)** -Diaminopimelate epimerase (*Proteus mirabilis*, AM942759; *P*. *luminescens*, plu4640)	94/97	186	6e-14
	***fabD* (-)** -Malonyl CoA-acyl carrier protein transacylase (*Yersinia enterocolitica*, YE1634; *P*. *luminescens*, plu2834)	78/90	312	5e-50
	***fld* (-)** -Flavodoxin/nitric oxide synthase (*P*. *luminescens*, plu0667)	67/79	126	1e-9
	***fmt*** **(-)** -Methionyl-tRNA formyltransferase (*S*. *proteamaculans*, Spro_4512; *P*. *luminescens*, plu4696)	64/84	189	2e-25
	***fuyA* (-)** -Probable siderophore receptor (*P*. *luminescens*, plu2316)	77/93	157	5e-14
	***gidA*** **(-)** -Glucose inhibited division protein A (*P*. *luminescens*, plu0049)	88/96	285	9e-45
	***glmU* (-)** -UDP-N-acetylglucosamine pyrophosphorylase (*P*. *luminescens*, plu0038)	97/100	109	6e-13
	***glnE* (-)** -Glutamate-ammonia-ligase adenylyltransferase for glutamine synthetase (*Yersinia pestis*, YPTS_3551; *P*. *luminescens*, plu3969)	97/97	105	7e-11
	***leuC*** **(-)** -3-isopropylmalate dehydratase (*Erwinia tasmaniensis*, ETA_30520)	87/93	142	3e-20
	***pheA* (-)** -Prephenate dehydratase / chorismate mutase (*Enterobacter* sp, Ent638_3077; *P*. *luminescens*, plu1265)	72/89	180	2e-23
	***purA*** **(-)** -Adenylosuccinate synthetase (*P*. *luminescens*, plu4577)	84/88	205	6e-19
	***spt/agt*** **(-)** -Serine:pyruvate/alanine:glyoxylate aminotransferase (*Synechococcus* sp, SynRCC307_1294)	66/84	128	5e-08
	***sucA* (-)** -Alpha-ketoglutarate dehydrogenase (*P*. *luminescens*, plu1430)	76/85	217	3e-36
	***tyrS*** **(-)** -Tyrosine-tRNA synthetase (*P*. *luminescens*, plu2596)	95/95	160	5e-23
**Unknown**	***XK_scots11* (+)** -Hypothetical protein (*X*. *nematophila*, XNC1_0479)	221/252	253	2e-86
	***XK_scots12* (+)** -Hypothetical protein (*X*. *nematophila*, XNC1_1281)	166/201	201	8e-53
	***XK_scots13* (+)** -Hypothetical protein (*Xenorhabdus bovienii*, XBJ1_2577)	213/232	233	3e-91
	***XK_scots14* (+)** -Hypothetical protein (*X*. *nematophila*, XBJ1_2660)	71/80	105	1e-21
	***XK_scots15* (+)** -Hypothetical protein (*Legionella pneumophila*, plpp0051)	67/92	144	2e-12
	***XK_scots16* (+)** -No similarity to known genes			
	***XK_scots17* (+)** -No similarity to known genes			
	***yqjE* (-)**–Putative inner membrane protein (*X*. *nematophila*, *yqjE*)	183/193	230	2e-81
	***XK_scots18*** **(-)** -Putative Rhs family protein (*X*. *nematophila*, XNC1_3569)	131/137	184	9e-58
	***XK_scots19*** **(-)** -Hypothetical protein (*X*. *nematophila*, XNC1_1649)	52/63	173	1e-10
	***XK_scots20* (-)** -Hypothetical protein (*X*. *nematophila*, XNC1_0557)	169/171	195	1e-81
	***XK_scots21* (-)** -Sulfatase domain protein (*X*. *nematophila*, XNC1_0750)	121/136	161	8e-45
	***XK_scots22*** **(-)** -Putative Maf-like protein (*X*. *nematophila*, XNC1_0787)	115/124	158	2e-46
	***XK_scots23*** **(-)** -Hypothetical protein (*X*. *nematophila*, XNC1_3728)	75/84	108	4e-27
	***XK_scots24*** **(-)** -Hypothetical protein (*X*. *bovienii*, XBJ1_1339)	79/91	116	7e-25
	***XK_scots25* (-)** -Hypothetical protein (*Methylobacterium populi*, Mpop_5457)	71/81	147	3e-17
	***XK_scots26* (-)** -No similarity to known genes			
	***XK_scots27*** **(-)** -No similarity to known genes			
	***XK_scots28*** **(-)** -No similarity to known genes			
	***XK_scots29*** **(-)** -No similarity to known genes			
	***XK_scots30* (-)** -No similarity to known genes			
	***XK_scots31* (-)** -No similarity to known genes			
	***XK_scots32* (-)** -No similarity to known genes			

Genes up- and down-regulated are indicated as **(+)** and **(-)**, respectively. Genes under the regulation category are regulatory genes involved in controlling the expression of one or more other genes. Genes underlined were also identified to be differentially regulated in response to *in vitro* stationary-phase starvation conditions.

### Significance of the differentially expressed genes in *X*. *koppenhoeferi*

Homology searches divided the identified genes into several functional groups (S3 Fig): cell surface structure, regulation, stress response, nucleic acid modification, transport, metabolism, and unknown transcripts. Specifically, for metabolic adaptation, gene *acnB* involved in both TCA cycle and glyoxylate pathway was up-regulated whereas *sucA* required in TCA cycle was down-regulated. Genes involved in amino acid metabolism (e.g. *pheA* required for aromatic amino acid biosynthesis) were down-regulated, and genes corresponding to nutrient or growth factor uptake (e.g. *potD*) were also down-regulated. For transport systems, genes involved in proton uptake were up-regulated (*kefC*) but those for proton export (*cyoD*, *nqrC* and *nqrF*) were down-regulated. Genes required for cell motility and biofilm formation were down-regulated (*pilT*, *flhA*, *ppiD*, *fld*, *glmU* and *tldD*). Other changes in bacterial gene expression included those (such as transcriptional regulators and transposases) involved in replication, transcription and translation processes. In addition to the genes with annotated functions, 9 transcriptional sequences were totally new sharing no similarity to any gene or protein in current GenBank database. These differentially expressed unknown sequences may be specific to *X*. *koppenhoeferi*—*S*. *scarabaei* association and further investigations may provide clues to the determinants of specificity in nematode-bacteria symbiosis.

### Specificity and signature of the identified *X*. *koppenhoeferi* genes in other bacteria

**Fig 1:**
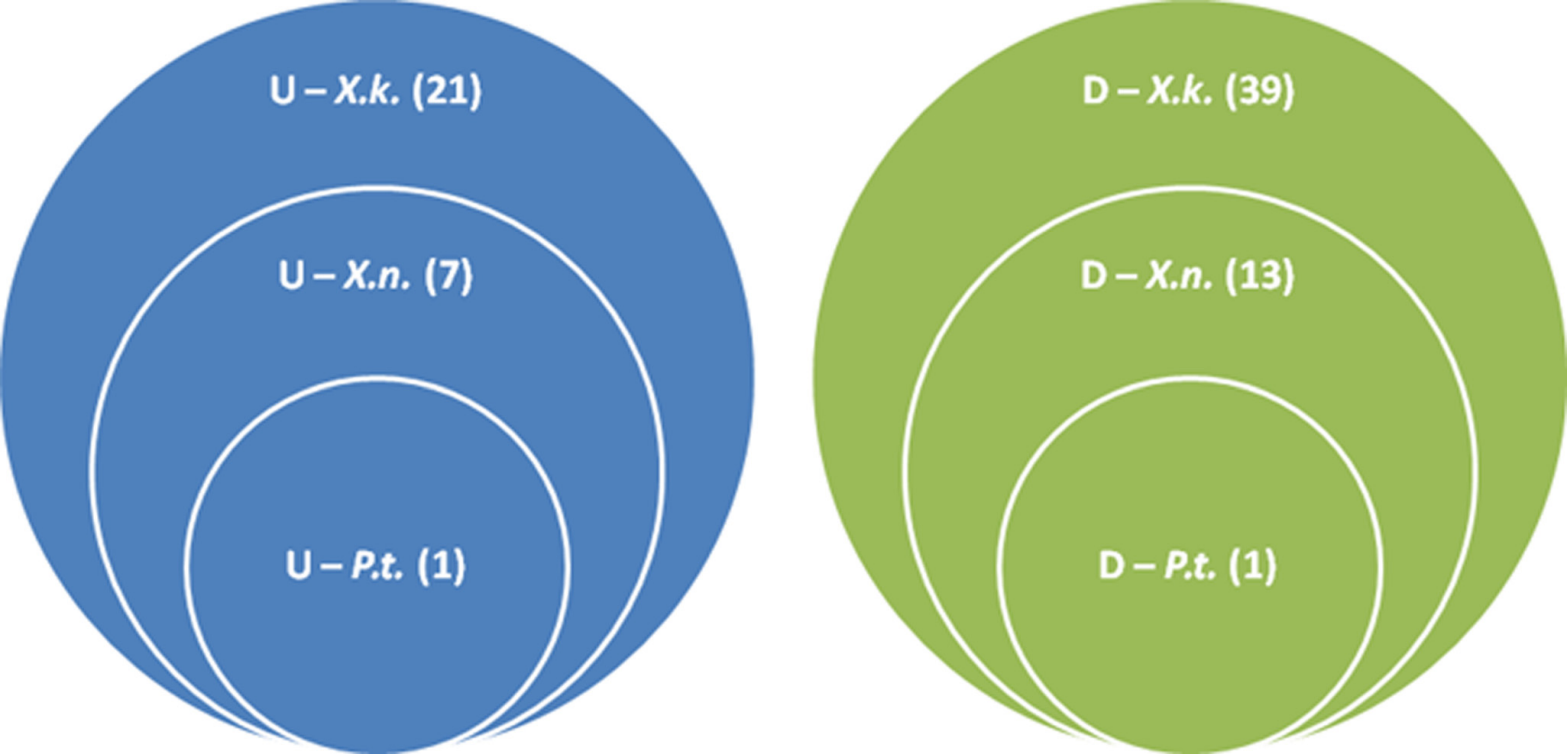
The number and distribution of SCOTS identified genes in *Xenorhabdus koppenhoeferi* during persistence in *Steinernema scarabaei*. The Venn diagram shows the proportion of up- (blue color) and down-regulated (olive green) genes unique to *X*. *koppenhoeferi* (*X*.*k*.) or common to the bacteria *Xenorhabdus nematophila* (*X*.*n*.) and *Photorhbdus temperata* (*P*.*t*.) during persistence in their nematode hosts.

Our previous study [27] identified 106 *P*. *temperata* genes differentially expressed during persistence in *H*. *bacteriophora* relative to growth in the nutrient medium. Comparative analysis with the 82 *X*. *koppenhoeferi* genes identified in this study showed that 27 out of the 106 and 29 out of the 82 gene sequences were specific to the respective bacterial genomes (Tables 2 and 3). Although the rest of the gene sequences were present in the genomes of both *P*. *temperata* and *X*. *koppenhoeferi*, only *secD* (down-regulated) and *xpsD* (up-regulated) had the same expression pattern in the two bacteria. More gene expression features were shared among *Xenorhabdus* species than those shared between *Xenorhabdus* and *Photorhabdus* (Fig 1). Out of the 82 genes identified in *X*. *koppenhoeferi*, 22 (8 up-regulated and 14 down-regulated) were found to be up-/down-regulated in the same direction in *X*. *nematophila* during persistence in *S*. *carpocapsae* (Table 4). The up-regulated genes included *flhC*, *hpcR*, *iscR*, *deaD*, *kefC*, *acnB*, *pta*, and *xpsD* and the down-regulated genes were *pilT*, *lepA*, *tldD*, *cutC*, *secD*, *accC*, *coaA*, *gidA*, *glmU*, *purA*, *sucA*, *yqjE*, *XK_scots21* and *XK_scots23*. Such shared expression signature was also verified via qPCR analysis of the relative changes in the gene expression in *X*. *nematophila* (S4 Fig).

**Table 2:** Genomic presence of the identified up-regulated *Xenorhabdus koppenhoeferi* sequences in other nematode-symbiotic bacteria.

Gene	*X*. *n*.	*X*. *b*.	*P*. *l*.	*P*. *a*.	Gene	*X*. *n*.	*X*. *b*.	*P*. *l*.	*P*. *a*.
*flhC*	+	+	+	-	*kefC*	+	+	+	+
*hpcR*	+	+	+	+	*ompX*	+	-	-	-
*iscR*	+	+	+	+	*pperX*	+	+	+	+
*lysR*	-	-	-	-	*aceE*	+	+	+	+
*grsD*	+	-	-	-	*acnB*	+	+	+	+
*deaD*	+	+	+	+	*pta*	+	+	+	+
*XK_scots01*	-	-	-	-	*xpsD*	+	+	-	-
*XK_scots02*	+	+	-	-	*XK_scots11*	+	+	+	+
*XK_scots03*	+	+	+	-	*XK_scots12*	+	+	-	-
*XK_scots04*	+	+	+	-	*XK_scots13*	-	+	-	-
*XK_scots05*	-	-	-	-	*XK_scots14*	+	-	-	-
*XK_scots06*	+	-	-	-	*XK_scots15*	-	-	-	-
*XK_scots07*	+	+	-	-	*XK_scots16*	-	-	-	-
*XK_scots08*	+	-	-	-	*XK_scots17*	-	-	-	-
*XK_scots09*	+	-	-	-					

The identified genes in this study were analyzed through blastn in NCBI to examine whether similar sequences are present in other bacteria. *X*. *n*.—*Xenorhabdus nematophila*; *X*. *b*.—*Xenorhabdus bovienii*; *P*. *l*.—*Photorhabdus luminescens*; *P*. *a*.—*Photorhabdus asymbiotica*.

**Table 3:** Genomic presence of the identified down-regulated *Xenorhabdus koppenhoeferi* sequences in other nematode-symbiotic bacteria.

Gene	*X*. *n*.	*X*. *b*.	*P*. *l*.	*P*. *a*.	Gene	*X*. *n*.	*X*. *b*.	*P*. *l*.	*P*. *a*.
*pilT*	+	+	+	+	*glmU*	+	+	+	+
*rhs*	+	-	-	-	*glnE*	+	+	+	+
*lepA*	+	+	+	+	*leuC*	+	+	-	-
*tldD*	+	+	+	+	*pheA*	+	+	+	+
*tyrR*	+	+	+	+	*purA*	+	+	+	+
*cutC*	+	+	-	-	*spt/agt*	-	-	-	-
*ppiD*	+	-	-	-	*sucA*	+	+	+	+
*heaP*	+	+	+	+	*tyrS*	+	+	+	+
*intB*	+	+	+	+	*asmA*	+	+	-	-
*rep*	+	+	+	+	*yqjE*	+	+	-	-
*ruvA*	+	+	+	+	*XK_scots10*	+	+	+	+
*xni*	+	+	+	+	*XK_scots18*	+	-	-	+
*cyoD*	+	+	+	+	*XK_scots19*	+	-	-	-
*flhA*	+	+	+	+	*XK_scots20*	+	+	-	-
*nqrC*	+	+	+	+	*XK_scots21*	+	+	+	+
*nqrF*	+	+	+	+	*XK_scots22*	+	+	+	+
*potD*	+	-	-	-	*XK_scots23*	+	+	-	-
*secD*	+	+	+	+	*XK_scots24*	-	+	-	-
*accC*	+	+	+	+	*XK_scots25*	-	-	-	-
*atoA*	-	-	-	-	*XK_scots26*	-	-	-	-
*coaA*	+	+	+	+	*XK_scots27*	-	-	-	-
*dapF*	+	+	+	+	*XK_scots28*	-	-	-	-
*fabD*	+	+	+	+	*XK_scots29*	-	-	-	-
*fld*	+	+	+	+	*XK_scots30*	-	-	-	-
*fmt*	+	+	+	+	*XK_scots31*	-	-	-	-
*fuyA*	-	-	+	+	*XK_scots32*	-	-	-	-
*gidA*	+	+	+	+					

The identified genes in this study were analyzed through blastn in NCBI to examine whether similar sequences are present in other bacteria. *X*. *n*.—*Xenorhabdus nematophila*; *X*. *b*.—*Xenorhabdus bovienii*; *P*. *l*.—*Photorhabdus luminescens*; *P*. *a*.—*Photorhabdus asymbiotica*.

**Table 4:** Gene expression signature shared between *X*. *koppenhoeferi* and *X*. *nematophila* during persistence in their respective nematode hosts *S*. *scarabaei* and *S*. *carpocapsae*.

Genes	Gene product	Identities	Expression
*flhC*	Flagellar transcriptional activator	97/110 (88%)	+
*hpcR*	Homoprotocatechuate degradation operon regulator	208/233 (89%)	+
*iscR*	Transcriptional regulator	148/166 (89%)	+
*deaD*	DEAD box family DNA helicase	237/268 (88%)	+
*kefC*	Potassium efflux system protein	110/125 (88%)	+
*acnB*	Aconitate hydratase	212/245 (87%)	+
*pta*	Phosphotransacetylase	175/199 (88%)	+
*xpsD*	Peptide synthetase	304/344 (88%)	+
*pilT*	Pilus retraction ATPase	139/156 (89%)	-
*lepA*	GTP-binding protein	238/245 (97%)	-
*tldD*	Suppressor of the carbon storage regulator	241/275 (88%)	-
*cutC*	Copper homeostasis protein	115/133 (86%)	-
*secD*	Protein-export membrane protein	206/212 (97%)	-
*accC*	Acetyl-CoA/biotin carboxylase	164/180 (91%)	-
*coaA*	Pantothenate kinase	197/219 (90%)	-
*gidA*	Glucose inhibited division protein	244/283 (86%)	-
*glmU*	UDP-N-acetylglucosamine pyrophosphorylase	102/105 (97%)	-
*purA*	Adenylosuccinate synthetase	164/181 (91%)	-
*sucA*	Alpha-ketoglutarate dehydrogenase	207/234 (88%)	-
*yqjE*	Hypothetical inner membrane protein	183/193 (95%)	-
*XK_scots21*	Hypothetical sulfatase domain protein	121/136 (89%)	-
*XK_scots23*	Hypothetical protein	74/85 (87%)	-

### Network analysis and biological interpretation of gene expression signature

**Fig 2:**
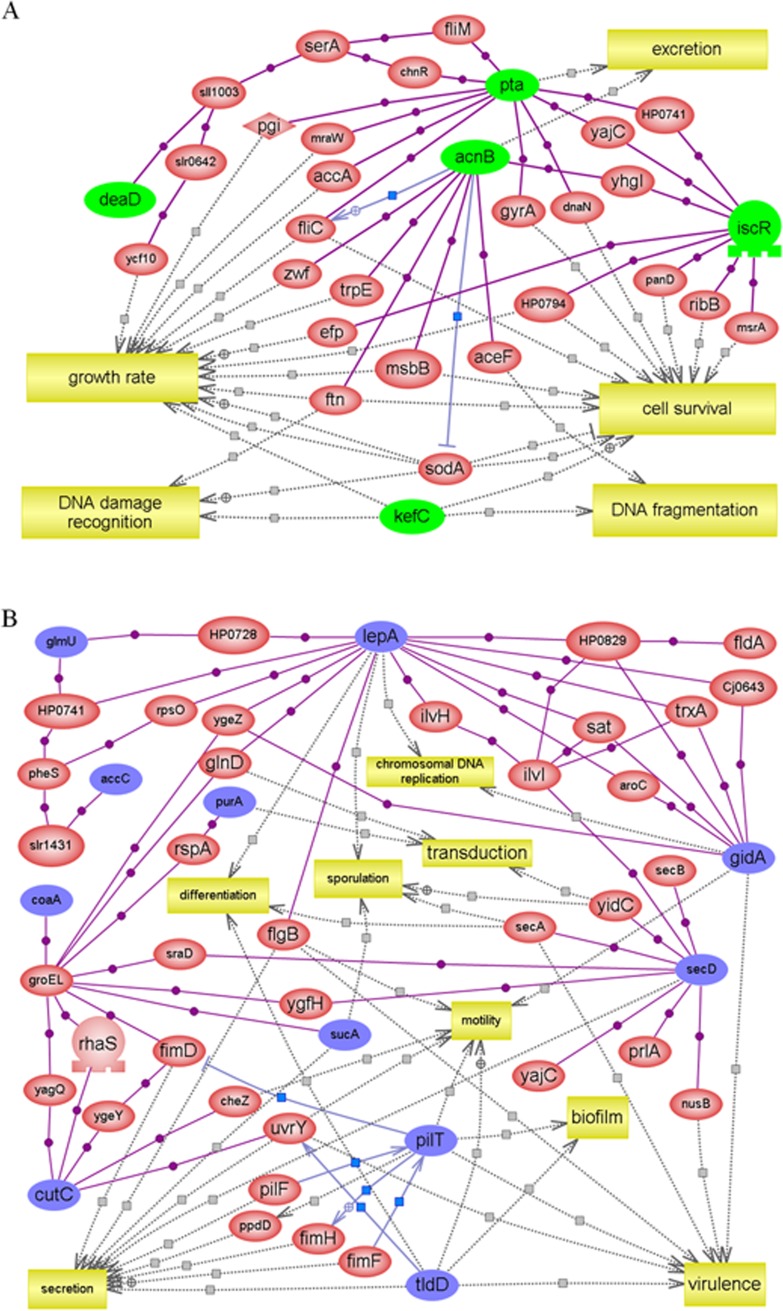
Pathway diagram of genes with expression patterns shared by *X*. *nematophila* and *X*. *koppenhoeferi* during persistence in their respective host nematode *S*. *carpocapsae* and *S*. *scarabaei*. Direct-connection networks for the genes with shared expression signatures were built in PathwayStudio by leveraging databases of currently published literatures. The genes are represented by red, blue or green ovals, where red ovals represent genes in the database of the PathwayStudio program with direct connection to the shared up- (green in panel A) and down-regulated (blue in panel B) genes identified in this study. Connecting lines between the gene symbols indicate interactions and different types of interactions are denoted by symbols on the lines. Purple square indicates binding; blue square, expression; grey square, regulation.

Using the PathwayStudio program, we were able to elucidate how the shared gene expression signatures between *X*. *nematophila* and *X*. *koppenhoeferi* while persisting in their respective host *S*. *carpocapsae* and *S*. *scarabaei* may influence other genes to drive or participate in the biological processes in the interactive network. The shared up-regulated genes were associated with five biological processes: "growth rate", "DNA fragmentation", "DNA damage recognition", "cell survival" and "excretion". The shared down-regulated genes were associated with eight processes: "DNA replication", "differentiation", "sporulation", "transduction", "motility", "biofilm", "secretion" and "virulence" (Fig 2). The up-regulated genes were mostly associated with bacterial survival and down-regulated genes were more related to bacterial virulence and active growth. These features shared by *X*. *koppenhoeferi* and *X*. *nematophila* were in accordance with our previous conclusions that *Photorhabdus* bacteria use a strategy of "cooperative endurance" by switching their metabolic activities to a survival mode in the nematode host [27]. As reducing metabolic activities appears to be a strategy shared by *Photorhabdus* and *Xenorhabdus* bacteria during living in their nematode hosts, we considered *acnB* (encoding a key enzyme in glyoxylate cycle) and *pta* (encoding a key enzyme in acetate switch) genes particularly interesting for further mutagenesis studies as they are directly involved in the metabolic adaptations.

### Impact of gene disruption on bacterial phenotype

**Fig 3:**
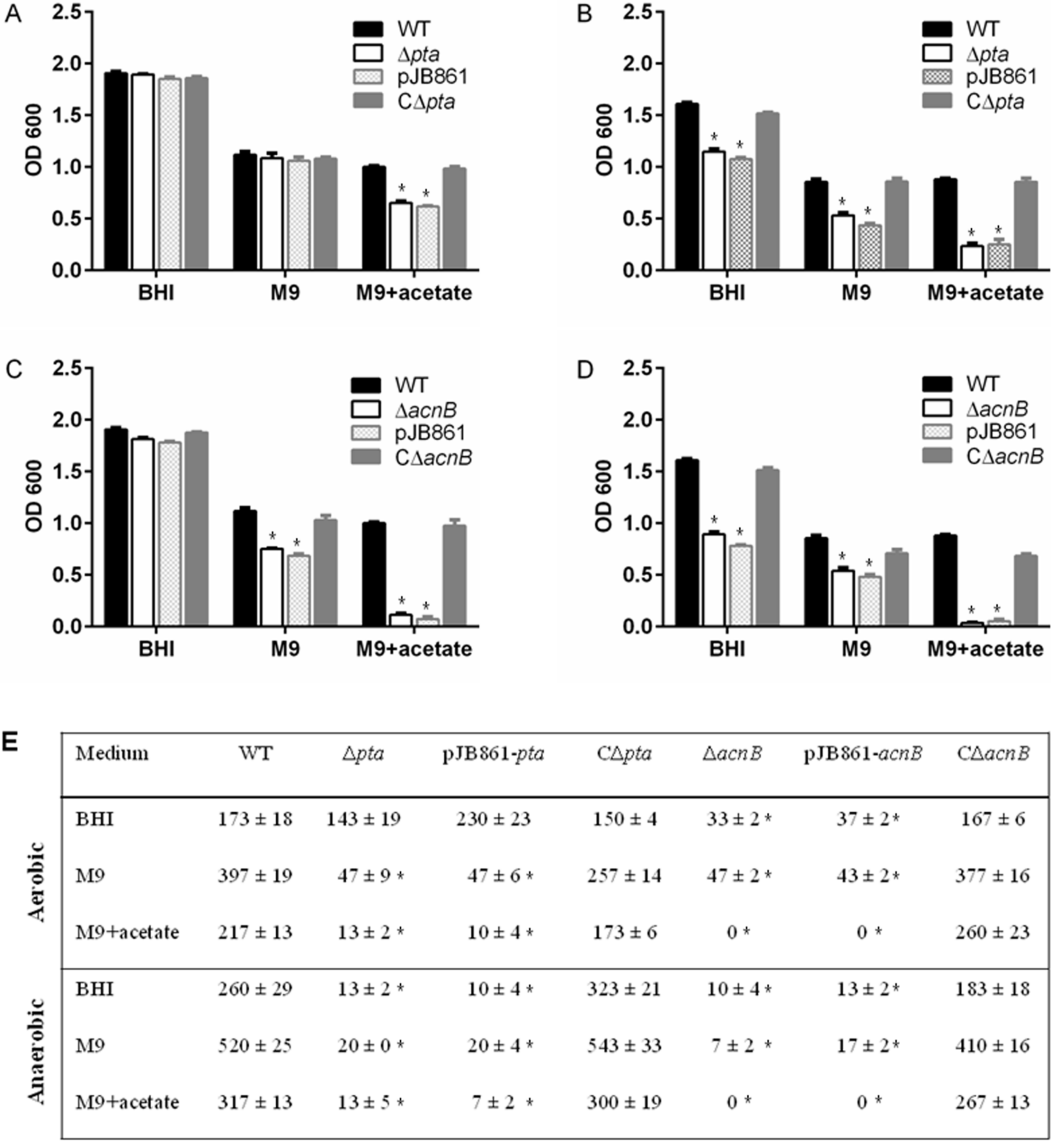
Growth and survival of *Xenorhabdus nematophila* wild-type, *pta* and *acnB* mutant cells *in vitro*. The bacteria were incubated aerobically or anaerobically at 25°C in the BHI broth, M9 minimal medium, and M9 minimal medium with acetate for up to 14 days. The relative extents of growth (A and C: aerobic growth; B and D: anaerobic growth) were recorded at 48 h by measuring OD 600 and viability (E) at 14 d by plating on BHI agar. WT represents the wild-type strain of *X*. *nematophila*, Δ*pta* denotes the mutant of the *pta* gene (encoding phosphotransacetylase), CΔ*pta* is the genetically complemented *pta*^+^ strain, Δ*acnB* denotes the mutant of the *acnB* gene (encoding aconitate hydratase), CΔ*acnB* is the genetically complemented *acnB*^+^ strain, and pJB861 indicates the mutants carrying the empty vector pJB861. Results (E) are shown as mean ± SEM from two independent experiments with three replicates per treatment, and the error bars (A, B, C, D) represent the standard error of the mean. Star symbol (*) indicates the statistical significance (p < 0.05) with comparison to the wild-type strain in each growth medium in the multiple *t* tests.

Colony morphology, motility, biofilm formation, cell growth, survival and viability were examined for phenotypic changes in *X*. *nematophila* mutants compared to the wild-type strain. Colony color, size, motility, and biofilm formation were not affected by the tested mutations (S2 Table). Compared with the wild-type strain, the *pta* mutant exhibited reduced growth and viability over time under anaerobic conditions while its aerobic growth and viability remained normal in BHI and glucose minimal medium (Fig 3). In acetate minimal medium, aerobic growth of the *pta* mutant within 48 h was similar to the wild-type strain, but its viability after 14 days was as low as the mutant cells incubated under anaerobic condition. Over a period of 14 days of anaerobic incubation, viable cell number of the *pta* mutant decreased dramatically compared to the wild-type cells during the same period (Fig 3). Bacterial growth and survival were also impaired by the mutation in *acnB* gene (Fig 3). Although the *acnB* mutant grew as well as the wild-type strain in BHI medium under aerobic condition, its anaerobic growth in BHI and growth in the glucose minimal medium were limited and the mutant failed to grow with acetate as a sole carbon source. Similar to the *pta* mutation, the *acnB* disruption also led to poor survival and viability of the mutant over time. The observed growth and viability defects in the mutants were fully restored by genetic complementation with trans-expression of the respective gene *pta* or *acnB* (Fig 3). Together, these results suggest that both *pta* and *acnB* are needed by *X*. *nematophila* to utilize acetate and survive better under anaerobic conditions.

### Effect of gene disruption on bacteria-nematode interaction

**Fig 4:**
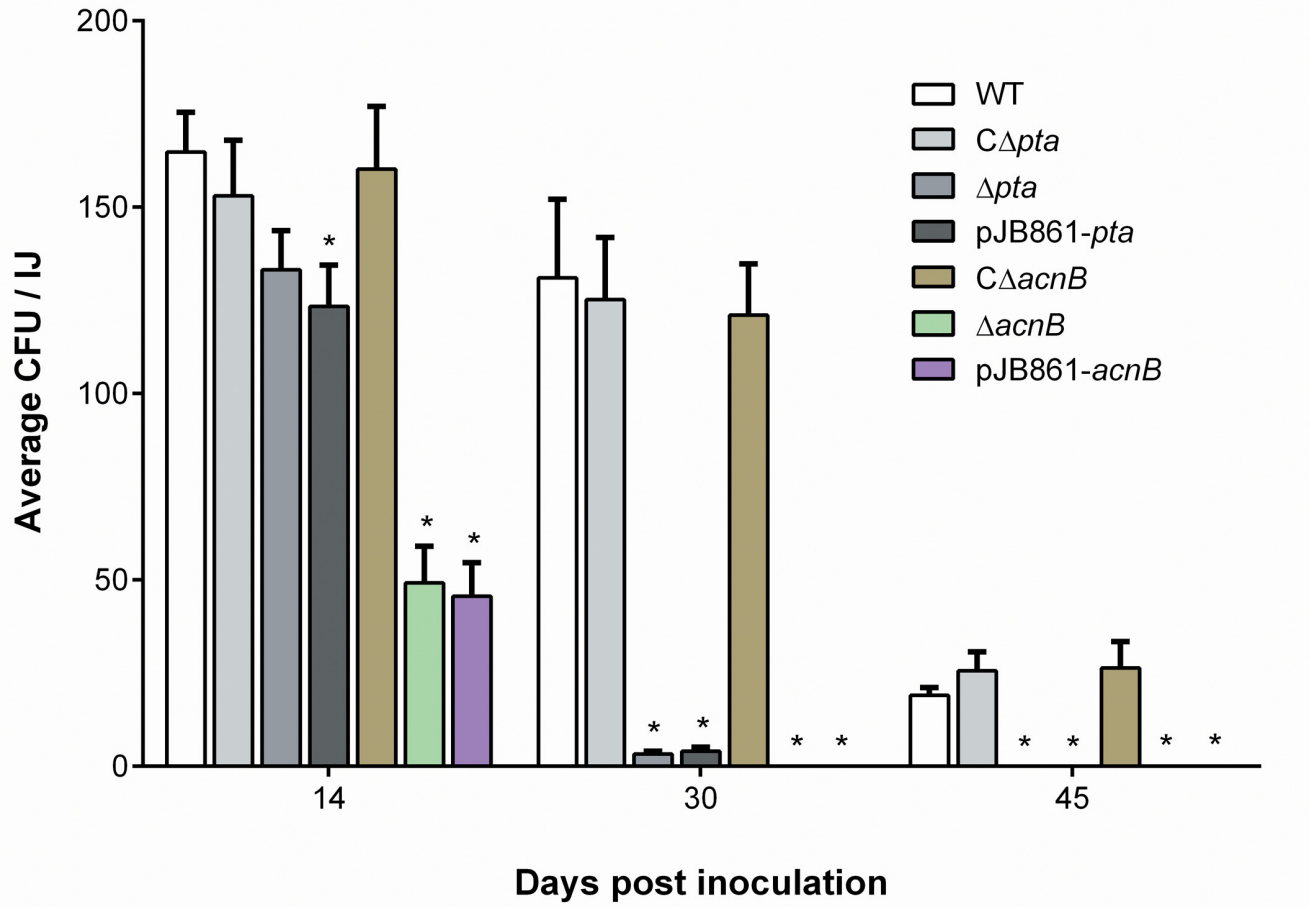
Persistence of the bacteria *Xenorhabdus nematophila* in the infective juvenile stage of nematode host *Steinernema carpocapsae*. The data shown are the mean (± SEM) number of the *pta* mutant (A: Δ*pta* denotes the *pta* mutant; CΔ*pta* is the genetically complemented *pta*^+^ strain; pJB861 indicates the *pta* mutant with the control vector pJB861), *acnB* mutant (B: Δ*acnB* denotes the *acnB* mutant; CΔ*acnB* is the genetically complemented *acnB*^+^ strain; pJB861 indicates the *acnB* mutant with the control vector pJB861) or wild-type (WT) bacterial cells persisting in an infective juvenile at 14, 30 and 45 days post-inoculation of the nematode eggs on the respective bacterial lawn. Star symbol (*) indicates the statistical significance (p < 0.05) with comparison to the wild-type strain in the multiple *t* tests.

Nematode growth and reproduction appeared normal on both *pta* and *acnB* mutants as compared with the wild-type *X*. *nematophila* cells. Adult nematodes developed in 4–5 days after inoculation of eggs on the bacterial lawns and the next generation infective juveniles developed within 2 weeks. However, ability of the bacteria to persist in the infective juvenile was drastically reduced in both *pta* and *acnB* mutants and the number of viable cells in the nematode decreased much more rapidly in the mutants compared with the wild type (Fig 4). The mean number of bacterial cells in a newly produced infective juvenile was slightly lower for the *pta* mutant but almost half for the *acnB* mutant, compared to the wild-type bacteria. Only 4 (±1) *pta* mutant cells and no *acnB* mutant cells per infective juvenile were detected 30 days post inoculation of the eggs, which were significantly lower than the number of wild-type cells (131 ± 21) retained by each infective juvenile (Fig 4). No *pta* mutant cells were detected in the infective juveniles 45 days post inoculation of the eggs in contrast to 23 ± 5 wild-type cells still retained per infective juvenile. These differences were significant (two-way ANOVA; p < 0.0001) for both the day and strain factors. Ectopic expression of the gene *pta* or *acnB* complemented the colonization defect in the respective mutants (Fig 4). These results suggest that both *pta* and *acnB* genes are required for *Xenorhabdus* cells to survive in the infective juvenile vesicle.

## Discussion

Understanding molecular basis of fitness traits is of fundamental importance in symbiosis studies. Our *in vivo* gene expression analysis indicates key molecular features adopted by *Xenorhabdus* bacteria while in mutualism with their nematode partner. Almost half of the genes differentially expressed by *X*. *koppenhoeferi* appear to be involved in adaptation to general starvation, and such a pattern has also been observed in *Photorhabdus* during their persistence in *Heterorhabditis* infective juveniles [27]. The starvation transcriptional profile could be a result of the bacterial response to nutrient deprivation or active down-regulation of metabolism to avoid over-taxing the nematode partner. In either scenario, it emphasizes that the bacteria tend to reduce their nutritional dependence on the nematode partner thus enhancing the couple's longevity during their search for a suitable insect host.

Our gene expression data suggest that the bacteria *X*. *koppenhoeferi* make large metabolic adaptations to persist in the nematode intestinal vesicle compared to growth in the nutrient medium. Unlike the utilization of pentose phosphate pathway by *P*. *temperata* [27], *Xenorhabdus* spp may enter into the glyoxylate pathway bypassing TCA cycle in the infective juvenile as indicated by the down-regulation of a key TCA cycle gene *sucA* and up-regulation of glyoxylate pathway gene *acnB* in both *X*. *koppenhoeferi* and *X*. *nematophila*. Also, the gene *coaA* encoding pantothenate kinase responsible for the biosynthesis of CoA, a coenzyme notable for its role in the oxidation of pyruvate in TCA cycle, was down-regulated in both *Xenorhabdus* bacteria. Instead, the CoA pool could be possibly maintained by recycling through the glyoxylate pathway [55]. In fact, metabolic change can be a critical component of survival responses and is often coordinated with the changes in lifestyles as cells must switch from a physiological state that permits rapid growth in the presence of abundant nutrients to one that enhances survival under famine [55,56]. In *P*. *luminescens*, the lifestyle shift was suggested to be triggered by a metabolic switch closely linked to the TCA cycle, but not by the well-known acetate switch [57]. It could be a different story in *Xenorhabdus* bacteria as indicated by the up-regulation of gene *pta* encoding phosphotransacetylase, a crucial enzyme in acetate switch, in both *X*. *koppenhoeferi* and *X*. *nematophila*. Previous studies suggest that the glyoxylate pathway allows some bacteria to grow and persist on acetate or other compounds that yield acetyl-CoA as a replenishing source for carbon in the synthesis of glucose and other important reactions [56,58]. Our mutagenesis experiments confirm that both *acnB* and *pta* genes are critical to *X*. *nematophila* for a prolonged growth with acetate *in vitro* and persistence in the infective juvenile of *S*. *carpocapsae*. Therefore, we suggest that the acetate switch most likely plays a major role in mediating lifestyle shifts in *Xenorhabdus* bacteria. The acetate switch has also been reported to affect the symbiosis of *Vibrio fischeri* with squid through a metabolic connection between acetate utilization and bacterial cell density [59].

Along with central metabolic pathways, the bacteria also appear to alter metabolism of other substances like fatty acids and amino acids. While the most important function of acetyl-CoA carboxylase is to provide the malonyl-CoA substrate for the biosynthesis of fatty acids through irreversible carboxylation of acetyl-CoA [60,61], down-regulation of its corresponding gene *accC* in both *X*. *koppenhoeferi* and *X*. *nematophila* may indicate the reduced demand for fatty acids by these bacteria during persistence in the nematode vesicle. In *P*. *temperata*, we discovered that up-regulation of homoserine biosynthesis gene *metL* was essential for bacterial persistence in its nematode partner [27]. In *X*. *nematophila*, threonine and methionine were found to be required for colonization of the vesicle of *S*. *carpocapsae* infective juveniles [24]. Down-regulation of lysine and leucine biosynthesis genes (*dapF* and *leuC*) in *X*. *koppenhoeferi* therefore may suggest that these two amino acids are either not needed by the bacteria for persistence in the nematode or are provided by the host nematode. But these two genes were not found to be down-regulated in *X*. *nematophila* during persistence in *S*. *carpocapsae*, reflecting the different requirements for amino acids in the two species. In *P*. *temperata*, aromatic amino acid biosynthesis is possibly reduced due to the down-regulation of gene *aroG* [27], and *X*. *koppenhoeferi* may share this feature as the necessary gene *pheA* is down-regulated. This is also another example of different requirements for amino acids between *Xenorhabdus* species since *pheA* gene was not detected to be down-regulated in *X*. *nematophila*.

In the *P*. *temperata*—*H*. *bacteriophora* interaction, intracellular acidification is important for the bacteria to persist in the nematode partner [27]. This intriguing cellular process seems to be also induced by *X*. *koppenhoeferi* in the host nematode as indicated by the up-regulation of proton importer gene (*kefC*) and down-regulation of proton exporter genes (*cyoD*, *nqrC* and *nqrF*). Notably, we found that *kefC* gene was also up-regulated in *X*. *nematophila* during persistence in its nematode partner *S*. *carpocapsae*. Studies of other bacteria [62–65] have suggested that intracellular acidification occurs due to the differential regulation of proton transporter genes. Cellular acidification has the potential to control cell growth and protect bacteria from electrophile toxicity as noted in *Escherichia coli* [66]. This process may benefit *Xenorhabdus* by inhibiting the cell growth during persistence in the nematode. Such an assumption is consistent with the finding that *Xenorhabdus* fitness is maximized when nematodes carry intermediate but not higher numbers of bacterial cells [28].

Besides cellular acidification, several other down-regulated genes may also indicate limited bacterial growth inside the nematode. Amongst these, down-regulation of the polyamine importer gene *potD* in *X*. *koppenhoeferi* may result in retarded bacterial growth considering the importance of polyamines in initiating and maintaining cell growth in both prokaryotes and eukaryotes [67]. Down-regulation of the gene *lepA* in both *X*. *koppenhoeferi* and *X*. *nematophila* may result in a decreased rate of protein synthesis because the elongation factor LepA increases the rate of protein synthesis under certain stress conditions [68]. As a null mutation of *gidA* in *E*. *coli* leads to a reduced growth rate [69], down-regulation of this gene in both *X*. *koppenhoeferi* and *X*. *nematophila* may also reduce their growth rate and maximize fitness in the nematode infective juvenile.

Biofilm formation is suggested to be crucial in *Photorhabdus* during persistence in *H*. *bacteriophora* infective juvenile [17,18,27,36], which is likely not the case in *Xenorhabdus* bacteria. A number of genes (*pilT*, *flhA*, *ppiD*, *fld*, *glmU*, *purA* and *tldD*) involved in biofilm formation or cell motility were down-regulated in *X*. *koppenhoeferi*, and four of them (*pilT*, *glmU*, *purA* and *tldD*) shared expression signature with *X*. *nematophila* during persistence in the host nematode. Among these genes, *fld* has been shown to be important in translocation of enteric bacteria during infection [70,71]; *glmU* activity has been reported to be required by *E*. *coli* for biofilm formation [72] which can be activated by *tldD* gene product [73]; *pilT*, *flhA* and *ppiD* genes are usually needed for surface motility and biofilm formation in gram-negative bacteria [74–76]. Purine biosynthesis has been previously speculated to play a key role in biofilm formation in non-streptococcal species [77], thus up-regulation of the purine biosynthesis gene in *P*. *temperata* (*purL*) [27,36] but down-regulation in *X*. *koppenhoeferi* and *X*. *nematophila* (*purA*) supports the different status of biofilm development in the two bacterial genera. In addition, down-regulation of a Type IV pilus gene in *X*. *koppenhoeferi* further points to the lack of biofilm development. Overall, our results suggest that *Photorhabdus* and *Xenorhabdus* have different requirement for biofilm formation during persistence in their host nematodes. This is interesting as the two bacteria also occupy different niches in their host nematodes: *Photorhabdus* colonizes the entire intestine of *Heterorhabditis* but *Xenorhabdus* is restricted to the specialized vesicle in *Steinernema*.

Expression of factors necessary to colonize and persist in the host is often orchestrated by complex regulatory networks. Changes in bacterial gene expression may be linked with differential regulation of factors involved in replication, transcription and translation processes. Up-regulation of gene *hpcR* which negatively regulates activity of aromatic catabolism [78,79] in both *X*. *koppenhoeferi* and *X*. *nematophila* indicates that aromatic catabolism may be reduced in *Xenorhabdus* bacteria during persistence in *Steinernema* infective juveniles. This is consistent with the identification of the aromatic amino acids biosynthesis gene *pheA* being down-regulated in *X*. *koppenhoeferi*. A similar pattern exists in *P*. *temperata* as well: the *padR* gene which encodes a negative regulator of aromatic catabolism was up-regulated and aromatic amino acid biosynthesis genes were correspondingly down-regulated during persistence in *H*. *bacteriophora* infective juveniles [27]. Another important regulator identified in this study is IscR, an iron-dependent transcriptional regulator for a host of genes in response to changes in cellular Fe-S cluster status [80]. Up-regulation of *iscR* in both *X*. *koppenhoeferi* and *X*. *nematophila* may suggest important biology of Fe-S cluster synthesis in *Xenorhabdus* in *Steinernema* infective juveniles. A previous investigation of *X*. *nematophila* showed that disruption of *isc* operon prevented colonization of *S*. *carpocapsae* nematodes [81]. In *E*. *coli*, IscR seems to be a key iron regulator to control biofilm formation in response to changes in cellular Fe-S homeostasis, and this regulation leads to decreased surface attachment and biofilm dispersal under iron-limiting conditions [82]. Studies on *Erwinia chrysanthemi* have also predicted the remarkable role of IscR in the colonization of bacteria in a wide array of plant species [83]. Among the other regulators shared by *X*. *koppenhoeferi* and *X*. *nematophila*, TldD suppresses the inhibitory activity of a carbon storage regulator [84,85]. The carbon storage regulator is believed to control a variety of physiological traits such as biofilm formation and regulation of 'switches' between different physiological states in many pathogenic bacteria [86]. Thus, down-regulation of *tldD* gene in both *X*. *koppenhoeferi* and *X*. *nematophila* may be a necessity for the carbon storage regulator to play a role in coordinating physiological traits that are relevant to lifestyle switching in *Xenorhabdus*.

A number of transposase linked genes were up- or down-regulated in *P*. *temperata* [27] and *X*. *koppenhoeferi* while persisting in their respective host nematode *H*. *bacteriophora* and *S*. *scarabaei*. In fact, differential expression of genes related to transposases might be particularly linked with stress responses in organisms [87]. Increase in transposition activity contributes to genetic variability possibly for better adaptation and survival of bacterial species under stressful conditions such as acid, reduced nutrients, etc. [88–90]. Generally, stress is recognized as any environmental change that reduces the fitness of an organism, and it can be further separated into two classes according to the response of the organism: those evoking a physiological response and those evoking a phenotypic and/or a genetic response [87]. In line with this, the bacteria are definitely stressed during persistence in the host nematode since the number of the colonizing bacteria is restricted and the bacteria change into smaller-size phenotype in the nematode [18]. Here, we speculate that differential expression of the transposition related genes may also contribute to genetic variability for better adaptation and survival of the symbiotic bacteria in response to the changed environment from active growth *in vitro* or in insect hemolymph to reduced growth in the enduring nematode infective juveniles. One of these genes, *deaD*, shares the expression signature between *X*. *koppenhoeferi* and *X*. *nematophila* while persisting in their host nematode. Notably, this gene has been suggested to be critical for bacteria to adapt to a variety of stresses and survive under severe conditions according to a study of *Caulobacter crescentus*, a bacterium with a remarkable ability to grow at low temperatures [91]. Compared to *P*. *temperata*, more transposition involved genes encoding insertion sequence elements, resolvases, helicases, and recombinases were found to be differentially regulated in *X*. *koppenhoeferi*, and such expression signature may be attributed to *Xenorhabdus* genomes which harbor larger numbers of IS elements [30]. While the relationship between the IS elements and the occurrence of genome rearrangements in *Xenorhabdus* is yet unexplored [30], the differential expression of a number of these elements in *X*. *koppenhoeferi* in this study suggests that some of these may be relevant to symbiosis and deserve additional investigation.

In conclusion, both *Photorhabdus* and *Xenorhabdus* appear to reduce their growth and metabolism while living in the host nematode, which is attributed to the cellular acidification through regulation of proton transport systems, the down-regulation of genes required for active growth, and the switching of pathways for adaptive metabolic changes. The glyoxylate pathway is likely preferred by *Xenorhabdus*, whereas *Photorhabdus* utilizes the pentose phosphate pathway. Although both bacteria retain the ability to produce flagella, biofilms appear to be developed only in *Photorhabdus*. Further, *X*. *koppenhoeferi* shows up-regulation of a smaller set of genes compared to *P*. *temperata*. We detected very little overlap in the gene expression profiles between the two bacterial genera but a considerable similarity between the two *Xenorhabdus* species. Such distinction of gene expression between the two bacterial genera supports the independent evolution of symbiosis in the two systems [8,9,12]. Overall, this study has generated new information on the transcriptional mechanisms by which bacteria persist in mutualism with their nematode partners. It provides a genetic basis for exploring the recently available *X*. *nematophila* and *Xenorhabdus bovienii* genomes as well as a useful paradigm for investigating other symbiotic systems. It should be noted that the present findings are based on the comparative study of bacterial transcriptional levels between the *in vivo* and *in vitro* states on a single time point–freshly-produced infective juveniles that are between 3–5 days old. Given the possibility of changes in gene expression over time in the enduring nematode infective juveniles, transcriptional profiles on more time points facilitated with additional functional analyses in the future would be useful for even a more comprehensive mechanistic understanding of the nematode-bacterial symbiotic interactions.

## Supporting Information

S1 Fig Rarefaction analysis curves demonstrating coverage of cDNA libraries for genes identified from bacteria *Xenorhabdus koppenhoeferi* during colonization of the nematode host *Steinernema scarabaei*. The redundancy (or the colony counts) of each identified gene was counted cumulatively and the library coverage was calculated using Analytic Rarefaction program.
(TIF)

S2 Fig Fold changes in the expression of selected *Xenorhabdus koppenhoeferi* genes identified by SCOTS in the nematode host *Steinernema scarabaei*. Quantitative real-time PCR was used to evaluate the expression level of the SCOTS identified *X*. *koppenhoeferi* genes during colonization in the nematode host relative to growth in the artificial media. Error bars indicate the standard error of mean from three replications.
(TIF)

S3 Fig Classification of SCOTS identified up- and down-regulated genes in *Xenorhabdus koppenhoeferi* during persistence in *Steinernema scarabaei*. The numbers of genes involved in cell surface (e.g. pilin and outer membrane protein), regulation (regulatory genes controlling the expression of one or more other genes), stress response, nucleic acid modification (e.g. transposase and recombinase), transport (e.g. proton transporter), intracellular metabolism, and genes with unknown function or without similarity to known genes are presented.
(TIF)

S4 Fig Fold changes in the expression of selected *Xenorhabdus nematophila* genes during colonization of the nematode host *Steinernema carpocapsae*. These genes were identified by SCOTS procedure to be similarly expressed in *X*. *nematophila* and *X*. *koppenhoeferi* during colonization of their respective host nematode relative to growth in the artificial media. The shared gene expression signature in *X*. *nematophila* was verified via the quantitative real-time PCR analysis. Error bars indicate the standard error of mean from three replications.
(TIF)

S1 Table Primer sequences used in this study. (DOCX)

S2 Table Phenotypic characters of the wild-type, *pta* mutant and *acnB* mutant strains of *Xenorhabdus nematophila*. (DOCX)
