# Supplementary figures and images for: Comparative Analysis of Xenorhabdus koppenhoeferi Gene Expression during Symbiotic Persistence in the Host Nematode
Source: PLoS One. 2016 Jan 8;11(1):e0145739. doi: 10.1371/journal.pone.0145739 (PMC4706420; doi:10.1371/journal.pone.0145739)

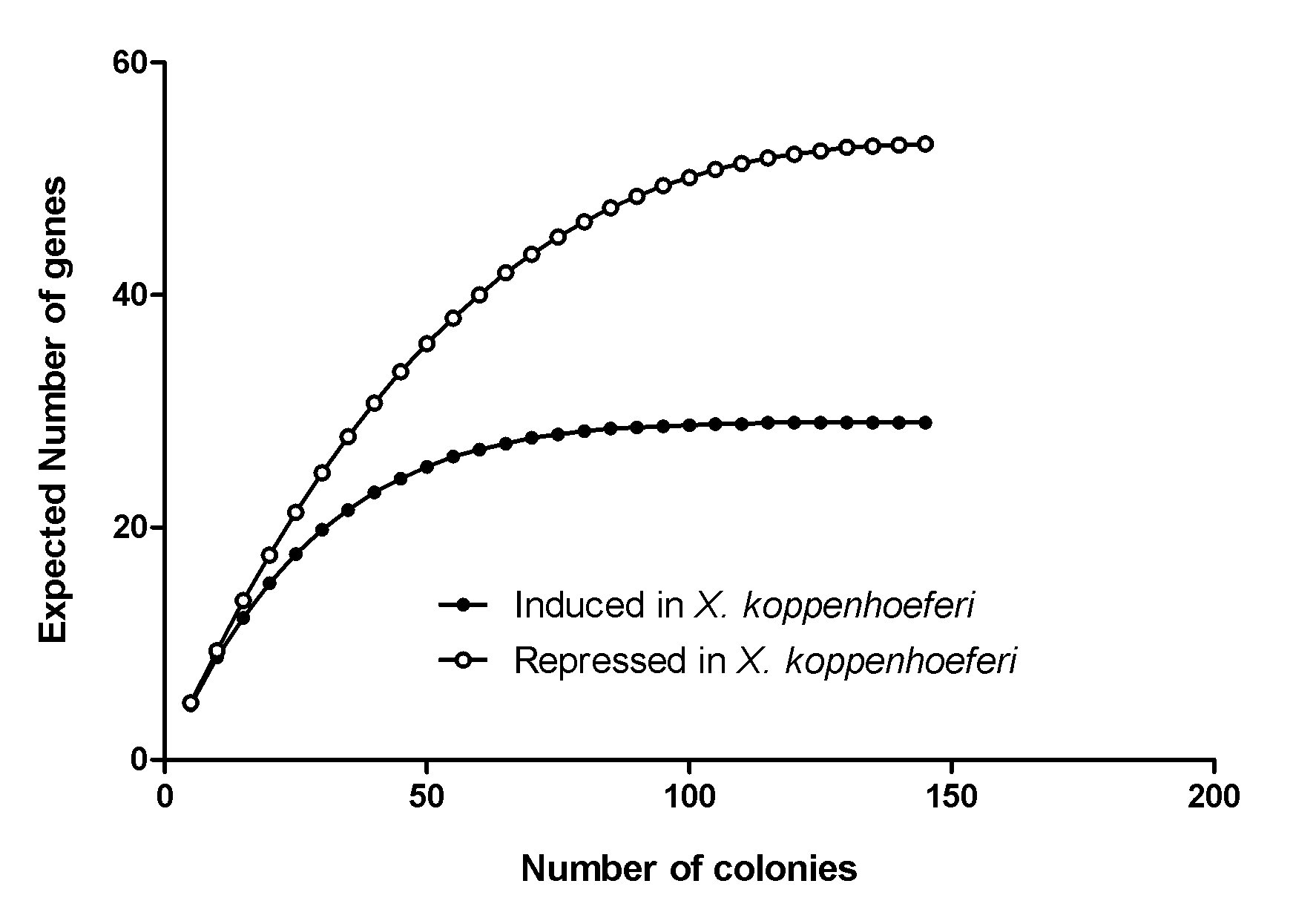

Supplement: S1 Fig — The redundancy (or the colony counts) of each identified gene was counted cumulatively and the library coverage was calculated using Analytic Rarefaction program. (TIF) [file pone.0145739.s001.tif]

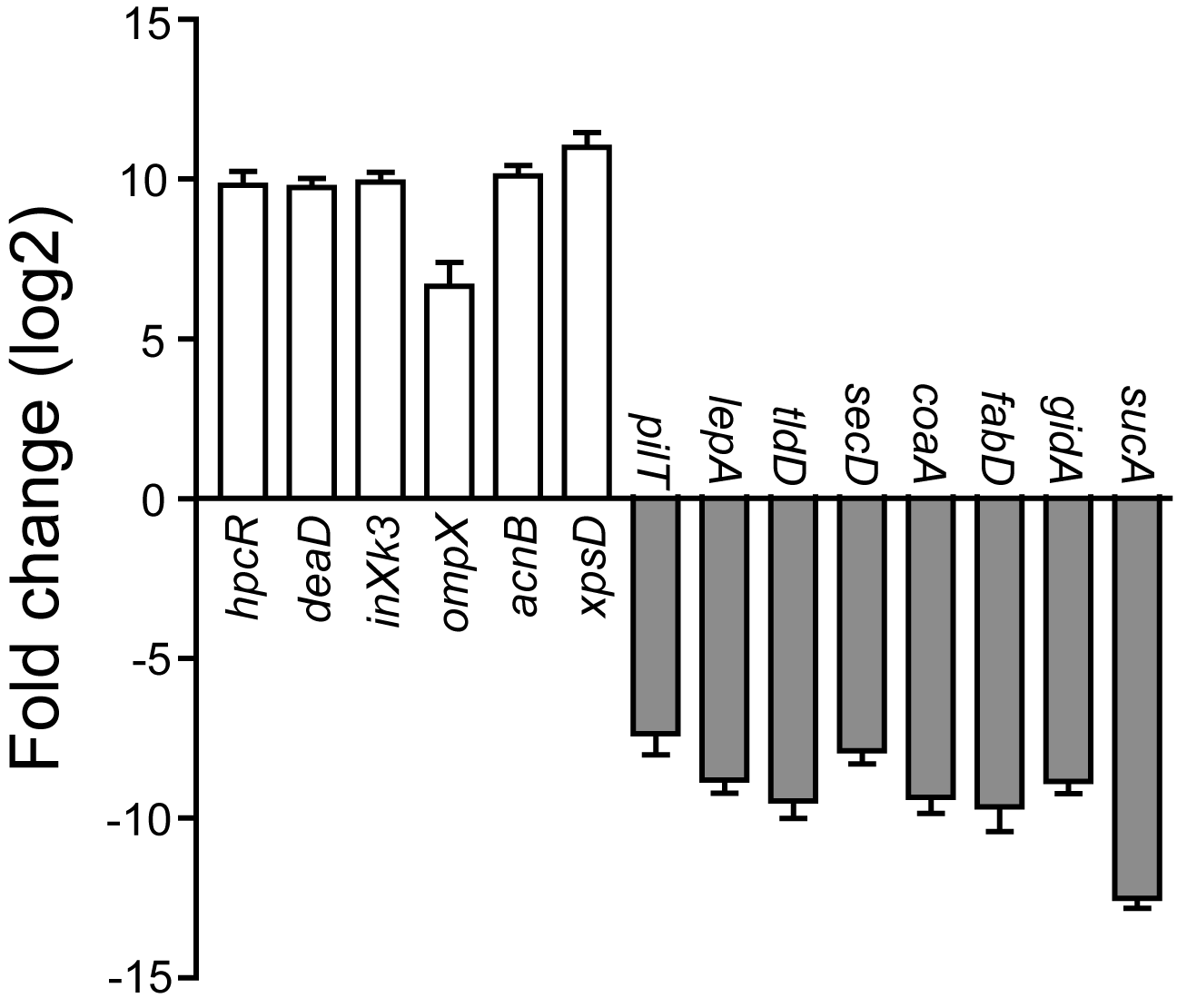

Supplement: S2 Fig — Quantitative real-time PCR was used to evaluate the expression level of the SCOTS identified X. koppenhoeferi genes during colonization in the nematode host relative to growth in the artificial media. Error bars indicate the standard error of mean from three replications. (TIF) [file pone.0145739.s002.tif]
