# Supplementary material for: Comparative Analysis of Xenorhabdus koppenhoeferi Gene Expression during Symbiotic Persistence in the Host Nematode
Source: PLoS One. 2016 Jan 8;11(1):e0145739. doi: 10.1371/journal.pone.0145739 (PMC4706420; doi:10.1371/journal.pone.0145739)
Supplement: S1 Table — (DOCX) [file pone.0145739.s005.docx]

**Table S1.** Primer sequences used in the study

| Primer | | Sequence (5' - 3') |
| --- | --- | --- |
| SCOTS procedure | | |
|  | ST0N | ATCCACCTATCCCAGTAGGAGNNNNNNNNN |
|  | ST0 | ATCCACCTATCCCAGTAGGAG |
|  | ST18N | GACAGATTCGCACTTAACCCTNNNNNNNNN |
|  | ST18 | GACAGATTCGCACTTAACCCT |
|  | ST110N | ATGCGAATCCAGACTGTAAGANNNNNNNNN |
|  | ST110 | ATGCGAATCCAGACTGTAAGA |
|  | 18SF | GGAATTGACGGAAGGGCACCA |
|  | 18SR | CCAGACAAATCGCTCCACCAAC |
|  | gyrAF | ACGCGACGGTGTACCGGCTT |
|  | gyrAR | GCCAGAGAAATCACCCCGGTC |
|  |  |  |
| RT-PCR analysis | | |
|  | acnB-F | TGTGCGCCTAACGATCCAGATGAT |
|  | acnB-R | ATGCTGATCCAATAACTTGCCCGC |
|  | coaA-F | AGTGGAAGGATTGTCCACACGAAC |
|  | coaA-R | CGCTTCTTCTGTAGTCAGCTTAGA |
|  | deaD-F | AATGAAGTGCAACTGCCTGATGCG |
|  | deaD-R | TTTGATAGATCACGGGTACGAGGTGC |
|  | fabD-F | TCTTGGTGAATACTCGGCACTGGT |
|  | fabD-R | AGCCAATAATGGCATACATCGCCC |
|  | gidA-F | ATGTTTACCTCCCGCGCTGAGTAT |
|  | gidA-R | AGCAGACGTTGACGTTCTTGTTCG |
|  | hpcR-F | GCAGCAATGGCGTATTATTCGCGT |
|  | hpcR-R | TACGCTGGTCGTTCATCGGTTTCA |
|  | lepA-F | GTGGCCGAACTAATAAACTGTCGC |
|  | lepA-R | TACCGTGGTCAATGTGGGCGATAA |
|  | ompX-F | ACAACATCCTGCAAGCAAACCGTG |
|  | ompX-R | ACCTAAACTGGCGAATGTGGTGGA |
|  | pilT-F | TACCAATCTGCCGCTGCTGTATCA |
|  | pilT-R | ACAGGAAGCGGGAAATCTACGACT |
|  | secD-F | CGTGAAGCGCGTTCTTATGCTGTT |
|  | secD-R | ATACGATCTGCGCCTTGACGTTGA |
|  | sucA-F | TCAACTTCGGCAGGAATGGAACTG |
|  | sucA-R | TGGCGTCCAATGGGATTTCACTCA |
|  | tldD-F | TCGTCCATTGGTACGTCTTTCCGT |
|  | tldD-R | ATTAATCAGTGCCATGCGAACCGC |
|  | tnXk3-F | ACCGACCGATTACGGTGATTGGAA |
|  | tnXk3-R | GGGATGGACTTTAATGTGGCTTGC |
|  | xpsD-F | TACTGCTGTTAACCCAGCACCACA |
|  | xpsD-R | TGCATATTGAATCGGCAGTGCAGG |
|  | 16S-F | AGCGCAACCCTTATCCTTTGTTGC |
|  | 16S-R | TCGCGAGGTCGCTTCTCTTTGTAT |
|  |  |  |
| Mutation construction and complementation | | |
|  | camR-P3 | TTGATCGGCACGTAAGAGGT |
|  | camR-P4 | AATTTCTGCCATTCATCCGC |
|  | pta-P1 | GTTCAGGTCAGGGAAGATGAAGA |
|  | pta-P2 | ACCTCTTACGTGCCGATCAACGTCTGGTAGAACTGCGTAAG |
|  | pta-P5 | GCGGATGAATGGCAGAAATTGTGTAACCACCGGTCAGTAAT |
|  | pta-P6 | AAGACGTGCTGATGGAAGAG |
|  | acnB-P1 | TCAAGGCATCGTCCCTAAAC |
|  | acnB-P2 | ACCTCTTACGTGCCGATCAAACGGAATATCTTCGCCCATAAA |
|  | acnB-P5 | GCGGATGAATGGCAGAAATTGACTGTGGAGAAGAAGGGTAAG |
|  | acnB-P6 | TGGTCATACAAGAGCCGATAAA |
|  | pta-FFL | CGGGATCCGCAACATGATCCTCAAACCG |
|  | pta-RFL | CGTCTAGAGAGTAAACCTACGAGCTCAACAG |
|  | acnB-FFL | CGGGATCCGTGCTGACAACGACCAATTAAC |
|  | acnB-RFL | CGTCTAGACAGAGGTAGTCCACAAACTGAC |
