# Supplementary material for: Comparative Analysis of Xenorhabdus koppenhoeferi Gene Expression during Symbiotic Persistence in the Host Nematode
Source: PLoS One. 2016 Jan 8;11(1):e0145739. doi: 10.1371/journal.pone.0145739 (PMC4706420; doi:10.1371/journal.pone.0145739)
Supplement: S2 Table — (DOCX) [file pone.0145739.s006.docx]

Table S2: Phenotypic characters of the wild-type, *pta* mutant and *acnB* mutant strains of *Xenorhabdus nematophila*

| Phenotypic traits | wild type | *pta* mutant | *acnB* mutant |
| --- | --- | --- | --- |
| Colony shape & size | Circular, small | Circular, small | Circular, small |
| Colony color | Light-yellow | Light-yellow | Light-yellow |
| Motility | 31.33 ± 1.45 | 31 ± 1.53 | 31.67 ± 1.45 |
| Biofilm formation | 0.43 ± 0.02 | 0.45 ± 0.01 | 0.42 + 0.02 |

The motility was measured as the size of colony (mm) after 24 h inoculation on 0.3% agar plate, and the data represent the mean (±SEM, n=3) value of the colony diameter. The biofilm formation was evaluated via crystal violet absorption at 600 nm after 24 h incubation of the cells in BHI media, and the data are the mean (±SEM, n=3) value of OD 600.
